# A di-electrophoretic simulation procedure of iron-oxide micro-particle drug attachment system for leukemia treatment using COMSOL software: a potential treatment reference for LMICs

**DOI:** 10.3389/fmedt.2023.1250964

**Published:** 2023-10-12

**Authors:** Henry Fenekansi Kiwumulo, Haruna Muwonge, Charles Ibingira, Michael Lubwama, John Baptist Kirabira, Robert Tamale Ssekitoleko

**Affiliations:** ^1^ Department of Medical Physiology, Biomedical Engineering Program, Makerere University, Kampala, Uganda; ^2^ Habib Medical School, Islamic University in Uganda (IUIU), Kampala, Uganda; ^3^ Department of Human Anatomy, Makerere University, Kampala, Uganda; ^4^ Department of Mechanical Engineering, Makerere University, Kampala, Uganda

**Keywords:** dielectrophoresis, leukemia, iron-oxide micro-particle, peak-to-peak voltage, modeling and simulation, COMSOL

## Abstract

**Background:**

Leukemia encompasses various subtypes, each with unique characteristics and treatment approaches. The challenge lies in developing targeted therapies that can effectively address the specific genetic mutations or abnormalities associated with each subtype. Some leukemia cases may become resistant to existing treatments over time making them less susceptible to chemotherapy or other standard therapies.

**Objective:**

Developing new treatment strategies to overcome resistance is an ongoing challenge particularly in Low and Middle Income Countries (LMICs). Computational studies using COMSOL software could provide an economical, fast and resourceful approach to the treatment of complicated cancers like leukemia.

**Methods:**

Using COMSOL Multiphysics software, a continuous flow microfluidic device capable of delivering anti-leukemia drugs to early-stage leukemia cells has been computationally modeled using dielectrophoresis (DEP).

**Results:**

The cell size difference enabled the micro-particle drug attachment to the leukemia cells using hydrodynamic focusing from the dielectrophoretic force. This point of care application produced a low voltage from numerically calculated electrical field and flow speed simulations.

**Conclusion:**

Therefore, such a dielectrophoretic low voltage application model can be used as a computational treatment reference for early-stage leukemia cells with an approximate size of 5 μm.

## 1. Introduction

Due to the prevailing limited data and resources, 80% of the 300,000 children diagnosed with cancer annually emerge from LMICs (1–3). Worse still, unlike liquid tumors like leukemia, much of the diagnosis and treatment best suits solid tumors which are easier to detect and diagnose. Liquid tumors, though also characterized by genetic abnormalities, may exhibit more complex molecular profiles, making targeted therapies more challenging to develop and implement. Additionally, evaluating treatment response in liquid tumors can be more complex, as circulating cancer cells may not be as easily quantifiable or measurable using traditional imaging methods (4). Leukemia encompasses various subtypes, each with unique characteristics and treatment approaches. The challenge lies in developing targeted therapies that can effectively address the specific genetic mutations or abnormalities associated with each subtype. Some leukemia cases may become resistant to existing treatments over time (5). This resistance can occur due to genetic mutations or changes within leukemia cells, making them less susceptible to chemotherapy or other standard therapies. Developing new treatment strategies or combination therapies to overcome resistance is an ongoing challenge.

Leukemia patients are treated using chemotherapy as the number one treatment method in LMICs. Such a treatment method renders poor selectivity, low treatment efficacy, hair loss, muscle weakening, general body weakness and high remission periods. These effects worsen as the tumor changes from solid to liquid nature which characterizes leukemia cancer cells (6–8). Additionally, this leukemia treatment is hampered more by the infrastructural challenges causing longer travel/wait times and assessment delays (9, 10–12). As compared to other treatment methods, many researchers have highlighted Modeling and Simulation Approaches (MSAs) as phenomenal approaches in targeting leukemia cells (13–16). These approaches have provided early detection techniques which are key factors in cancer treatment hence leading to long-term cancer survival for patients from the western world (17–21). Although many of these approaches involve *in vitro* and *in vivo* tools, computational tools have also emerged as preparatory tools capable of providing deeper insight than *in vitro*/*in vivo* tools (22, 23). Additionally, such computational models can easily provide a stepping platform for LMICs with limited data and therapeutic results (24).

In comparison to the proposed computational DEP approach, several leukemia treatment approaches that have been used include the following; Targeted Therapies (25)—These therapies focus on targeting specific molecules involved in leukemia growth and survival. Tyrosine kinase inhibitors (TKIs) have been used for certain types of leukemia, such as chronic myeloid leukemia (CML). Imatinib, dasatinib, and nilotinib are examples of TKIs that have shown promising results. Immunotherapy (26–28)—Chimeric Antigen Receptor (CAR) T-cell therapy has gained attention for the treatment of certain leukemias. CAR T-cell therapy involves modifying a patient's own immune cells to target and kill leukemia cells. Approved therapies like Kymriah and Yescarta have shown remarkable success in treating certain types of leukemia and lymphoma. Stem Cell Transplantation (29)—Allogeneic stem cell transplantation (bone marrow transplant) remains a crucial treatment option for many leukemia patients. Advances in this field include better matching techniques, reduced-intensity conditioning regimens, and improved post-transplant care. New Drug Approvals (30–31)—New drugs continue to be developed and approved for different types of leukemia. Venetoclax, for instance, has shown promise in treating certain cases of chronic lymphocytic leukemia (CLL) and acute myeloid leukemia (AML). Gene Editing (32)—Emerging technologies like CRISPR-Cas9 hold potential for treating leukemia by targeting and modifying specific genes in leukemia cells. Combination Therapies (33–35)—Researchers are exploring the use of combination therapies that involve different drugs or treatment approaches to improve outcomes and reduce resistance. Minimal Residual Disease (MRD) Monitoring (36, 37)—Advances in MRD detection allow for more sensitive monitoring of treatment response. This helps doctors assess the effectiveness of treatment and make informed decisions about further interventions. Precision Medicine (38–40)—As our understanding of the genetic and molecular basis of leukemia improves, personalized treatment plans based on a patient's specific genetic profile are becoming more common. Supportive Care (41, 42)—Improved supportive care measures, including management of side effects and infections, have led to better outcomes and quality of life for leukemia patients undergoing treatment.

Modeling and simulation using software like COMSOL can play a significant role in providing treatment reference models for leukemia. By using COMSOL software, researchers can create computational models that simulate the behavior of leukemia cells, the interaction with the immune system, and the impact of various treatment options. These models can help deepen our understanding of the disease, its progression, and the underlying mechanisms (43–46). COMSOL software allows for the creation of patient-specific models based on individual characteristics such as genetic information, medical history, and diagnostic test results. These models can be used to predict the response of a specific patient to different treatment strategies, helping doctors make informed decisions and design personalized treatment plans. Simulating the effects of different drug compounds on leukemia cells using COMSOL can aid in drug development. Researchers can test virtual compounds in silico, analyze their interactions with specific cellular targets, and predict their efficacy. This approach can help identify potential therapeutic agents and optimize drug dosages before moving to *in vitro* or clinical trials. Once a treatment plan is initiated, COMSOL can assist in monitoring the patient's response to therapy. By integrating real-time patient data with the computational model, healthcare providers can track the progression of leukemia, assess treatment effectiveness, and predict potential relapses. This information can guide treatment adjustments and optimize patient outcomes. COMSOL can facilitate the design and execution of virtual clinical trials. Instead of relying solely on costly and time-consuming traditional trials, researchers can simulate the effects of different treatment protocols on a large virtual patient population. This approach enables the evaluation of treatment strategies more efficiently, potentially accelerating the development and approval of new therapies. While using COMSOL, DEP where polarizable particles experience a force when exposed to a non-uniform electric field can be utilized to manipulate and separate cells or particles based on their electrical properties, such as their dielectric properties or surface charge (47, 48). In the context of leukemia treatment, microfluidic channels with integrated DEP systems could potentially offer several benefits. For example, DEP can be used to isolate and separate leukemia cells from blood samples, allowing for the detection and analysis of circulating tumor cells. This could aid in the diagnosis and monitoring of leukemia progression. Additionally, DEP-based microfluidic systems could enable the precise manipulation and positioning of cells, facilitating various treatment strategies. For instance, the targeted delivery of therapeutic agents to leukemia cells or the sorting of different cell populations based on their response to specific treatments. The systematic nature and cost-effectiveness of computational modeling and simulation has facilitated the understanding of several cancer-related therapies. Experimental designs are not only time-consuming, but they are also cumbersome and expensive as compared to computational models. Therefore, computational models might assist in analyzing the different leukemia cancer states to reduce the burden posed by the experimental approaches (49). Our group recently published a review showing a technological advancement in leukemia treatment concerning MSAs for HICs. This review characterized several computational models using various software platforms and revealed a gap in the usage of such models to enhance leukemia treatment in LMICs (50). Modeling and simulation using DEP is among the options that have revolutionized therapy. There is always a need to observe a specific voltage threshold while applying the DEP fields. This is because cell membrane permeabilization only occurs at a critical threshold that can prevent irreversible damage to the normal cells (51). Dielectrophoretic field parameters should therefore be adapted to each leukemic cell size to preserve the viability of the normal cells. Table 1 presents a summary of different dielectrophoretic models with their respective threshold voltages and therapeutic use.

**Table 1:** Micro-fluidic models using either dielectrophoretic or constant voltage potentials and their corresponding therapeutic uses.

Voltage potential type used	Potential therapeutic study	Voltage potential	Model output	Reference
Dielectrophoretic potential	Delivery of bioactive molecules, including Dexamethasone, from conductive polymers	−0.8 V to 1.4 V	Self-adjusting Dexamethasone drug release system for more than 3 weeks	Carli et al. (52)
Constant potential	The drug loading capacity of polypyrrole nanowire network for controlled drug release	−0.7 V for 30 min	Cyclic voltametric drug release model	Jiang et al. (53)
Dielectrophoretic potential	Carbon nanotube (CNT) for conducting polymer composite electrodes for drug delivery applications	0.3 V, −0.5 V, −0.7 V and −0.9 V for 2 min	A single walled CNT with increased drug release from 1.4,126–1.8864 mg/cm	Xiao et al. (54)
Dielectrophoretic potential	Investigate the active ionic liquids that can be used for polypyrrole electrosynthesis in controlled drug delivery	0.6 V, 1.2 V, or 1.5 V for 2h	Increased drug release rates with a negative potential increase and anionic nature	Carquigny et al. (55)
Dielectrophoretic potential	Drug release study pattern from a polypyrrole microchip	0.5 V, −0.8 V, and −1.0 V for 24 h	Implantable drug release microchip based on polypyrrole	Ge et al. (56)
Constant potential	Generates polymeric nanostructures in a 2D space to be used as conductive polymers	3 V for a maximum of 150s	A conductive polymeric nanostructure for use in 2D space	Dallas and Georgakilas (57)
Constant potential	A redox chemistry method for drug delivery and sensing while using electroactive polymers	−0.7 V for 700 s	First time quantifiable self regualating ATP release from polymers	Pernaut and Reynolds (58)
Dielectrophoretic potential	Polyaniline filaments used in Mesoporous channel host conduction	0.0 V after 10 min and then −0.6 V after 20min	Design of conductive filaments of polyaniline at an absorption frequency of 2.6 GHz	Wu and Bein (59)
Dielectrophoretic potential	Controlled release of heparin from polypyrrole -poly(vinyl-alcohol) assembly by electrical stimulation	Electric current pulses ranging from 0 mA to 3.5 mA	Developed a surface modification technique for heparin release	Li et al. (60)
Dielectrophoretic potential	Potential application method for controlled synthesis of polymers involved in micro/nanostructures	−0.6 V to 0.9 V	Various potential applications of micro/nanostructures as conducting polymers	Bajpai et al. (61)
Constant potential	Incorporation of sulphonated cyclodextrins into polypyrrole: an approach for the electro-controlled delivering of neutral drugs	−0.5 V for 600 s	An electro-approach for delivering neutral drugs into polypyrrole	Bidan et al. (62)
Dielectrophoretic potential	Electrochemical growth of polypyrrole microcontainers	−0.6 to 0.9 V	Designed high film/electrolyte double layer capacitive charges on the polypyrrole films	Qu et al. (63)
Dielectrophoretic potential	Facile fabrication of polymer and carbon nanocapsules using polypyrrole core/shell nanomaterials	0.7 V to −0.5 V with a gap of 14 min	Fabricated core/shell nanomaterials using polypyrrole	Jang et al. (64)
Dielectrophoretic potential	Polymer nanostructures and their applications in conducting biosensors	−1.2 V to 0.4 V	Conducting biosensors designed from polymer nanostrctures	Xia et al. (65)
Constant potential	Electrically controlled drug delivery studies from biotin-doped conductive polypyrrole	−0.5 V for 12 min	An electrically controlled drug delivery system from biotin-doped conductive polypyrrole	George et al. (66)
Dielectrophoretic potential	Electrochemically enhanced peptide-directed assembly of functional supramolecular polymers for enhanced drug delivery	−0.8 V to 1 V	Electrically triggered drug release from molecular tongue-twisters	Hardy et al. (67)
Dielectrophoretic potential	Ultra-low-voltage triggered the release of an anti-cancer drug from polypyrrole nanoparticles	−0.8 V to 0.4 V for 15 min	Designed an Ultra-low-voltage trigger for the release of an anti-cancer drug from polypyrrole nanoparticles	Samanta et al. (68)
Dielectrophoretic potential	Development of a controlled release system for risperidone using polypyrrole: mechanistic studies	−0.6 V to 0.6 V for 120 s	Designed a controlled release system for risperidone using polypyrrole	Svirskis et al. (69)
Dielectrophoretic potential	Physical and performance evaluation of polypyrrole drug delivery systems	−0.6 V to 0.6 V at 0.5 Hz	An electrical risperidone drug delivery implant system from polypyrrole	Svirskis et al. (70)
Dielectrophoretic potential	Electrochemical release of acetylcholine from supercritical carbon dioxide (scCO_2_)polymer films	1 V to −1 V for 24 h	An electronically triggered release model of acetylcholine from scCO_2_ polymer films	Löffler et al. (71)
Constant potential	Drug delivery study of Micro/nanostructures as conducting polymers	0.5 V for the 30 s	Micro/nanostructures for drug delivery through conducting polymers	Uppalapati et al. (72)
Dielectrophoretic potential	Design study of a nanostructured sustainable platform in energy applications for conducting polymers	0.4 V, 0.5 V or 0.6 V for 20 min	A nanostructured sustainable platform used in energy applications for conducting polymers	Ghosh et al. (73)

Unfortunately, most of these models are in-vitro using polymers that would require dedicated resources, specialized skills, and more time to implement. Such challenges have motivated our work to come up with a novel computational model that can provide useful insights and formulations for leukemia treatment in LMICs.

## 2. Methods

By exploiting the fact that the smallest leukemia cell can be around 5 micrometers in size (74) and can easily be seen by light microscopes (75) commonly used in LMICs, it is possible to provide a treatment reference for such early-stage leukemia cells. This can be through simulating and studying size-based attachment of anti-cancer microdrugs to the tiniest leukemia cells using a DEP force. When the electric field is computed in the frequency domain, the dielectrophoretic force feature adds contribution (Equation 1) to the total force exerted on the particles. Both leukemia and the iron-oxide particles onto which the drug is conjugated are assumed to be spherical. The following parameter values are additionally assumed while simulating the drug attachment as shown in Table 2 (48),

(1) $$\mathrm{Fdep}\;=\;2\pi r_{p}^{3}\varepsilon_{0}\;\;\mathrm{real}\;\;(\varepsilon_{r}^{*})\;\;\mathrm{real}\left(\frac{\varepsilon_{r,p-}^{*}\varepsilon_{r}^{*}}{\varepsilon_{r,p}^{*}+2\varepsilon_{r}^{*}}\right)\Delta [E_{\mathrm{rms}}{]}^{2}$$

**Table 2:** Parameter values used in the simulation.

Parameter description	Parameter value	Reference
Electric field frequency	100 kHz	Piacentini et al. (48), Egger and Donath (76), Park et al. (77)
Buffer medium conductivity	55 ms/m	Piacentini et al. (48)
Buffer relative permittivity	80	Piacentini et al. (48)
Buffer density	1,000 kg/m^3^	Piacentini et al. (48)
Buffer dynamic viscosity	0.001 Pa.s	Piacentini et al. (48)
Particle density (leukemia cells and the drug particles)	1,050 kg/m^3^	Egger and Donath (76)
Leukemia cell diameter	5 μm	Hao et al. (74)
Iron-oxide particle diameter	1.8 μm	Piacentini et al. (48)
Leukemia cell conductivity	0.31 s/m	Piacentini et al. (48), Egger and Donath (76), Park et al. (77)
Iron-oxide particle conductivity	0.25 s/m	Piacentini et al. (48), Egger and Donath (76), Park et al. (77)
Leukemia relative permittivity	59	Piacentini et al. (48), Egger and Donath (76), Park et al. (77)
Iron-oxide particle relative permittivity	50	Piacentini et al. (48), Egger and Donath (76), Park et al. (77)
Leukemia shell electrical conductivity (antibody)	1 μs/m	Piacentini et al. (48)
Drug shell electrical conductivity (antigen)	1 μs/m	Piacentini et al. (48)
Leukemia shell relative permittivity	4.44	Egger and Donath (76)
Iron-oxide particle shell relative permittivity	6	Egger and Donath (76)
Leukemia shell thickness	9 nm	Piacentini et al. (48)
Iron-oxide particle shell thickness	8 nm	Piacentini et al. (48)

where

• *r*_p _= radius of a spherical particle in the field,
• $\varepsilon_{0}$_ _=_ _8.854187817·10−12 F/m is the vacuum permittivity,
• $\varepsilon_{r}^{*}$_ _=_ _complex relative permittivity of the buffer,
• $\varepsilon_{r,p}^{*}$_ _= complex relative permittivity of the particle,
• E_rms _= root mean square electric field.

The shell sub-node is used to model the drugs that are conjugated onto the micro iron-oxide particles. This sub-node is added to the dielectrophoretic force node to model the dielectrophoretic force on particles with thin dielectric shells. The electrical conductivity of the drug is different from the electrical conductivity of the iron-oxide particle to easily simulate the drug attachment application. When computing the dielectrophoretic force, the complex permittivity $\left(\varepsilon_{r,p}^{*}\right)$ of the particle is replaced by the equivalent complex relative permittivity $\left(\varepsilon_{eq}^{*}\right)$ of a homogeneous particle comprising both the shell (drug) and the interior of the particle. Therefore, the equivalent relative permittivity $\left(\varepsilon_{\mathrm{eq}}^{*}\right)$ in Equation 2 substitutes for $\left(\varepsilon_{r,p}^{*}\right)$ in Equation 1 to compute the DEP force (48).

(2) $$\varepsilon_{eq}^{*}=\varepsilon_{s}^{*}\frac{{\left(\frac{r_{0}}{r_{i}}\right)}^{3}+2\left(\frac{\varepsilon_{r,p}^{*}-\varepsilon_{r,s}^{*}}{\varepsilon_{r,p}^{*}+2\varepsilon_{r,s}^{*}}\right)}{{\left(\frac{r_{0}}{r_{i}}\right)}^{3}-\left(\frac{\varepsilon_{r,p}^{*}-\varepsilon_{r,s}^{*}}{\varepsilon_{r,p}^{*}+2\varepsilon_{r,s}^{*}}\right)}$$

where

$r_{0}$ and $r_{i}$ = outer and inner radii of the shell, respectively,
$\varepsilon_{r,p}^{*}$ = complex relative permittivity of the particle,
$\varepsilon_{r,s}^{*}$ = complex relative permittivity of the outer shell.

## 3. Results

**Figure 1:**
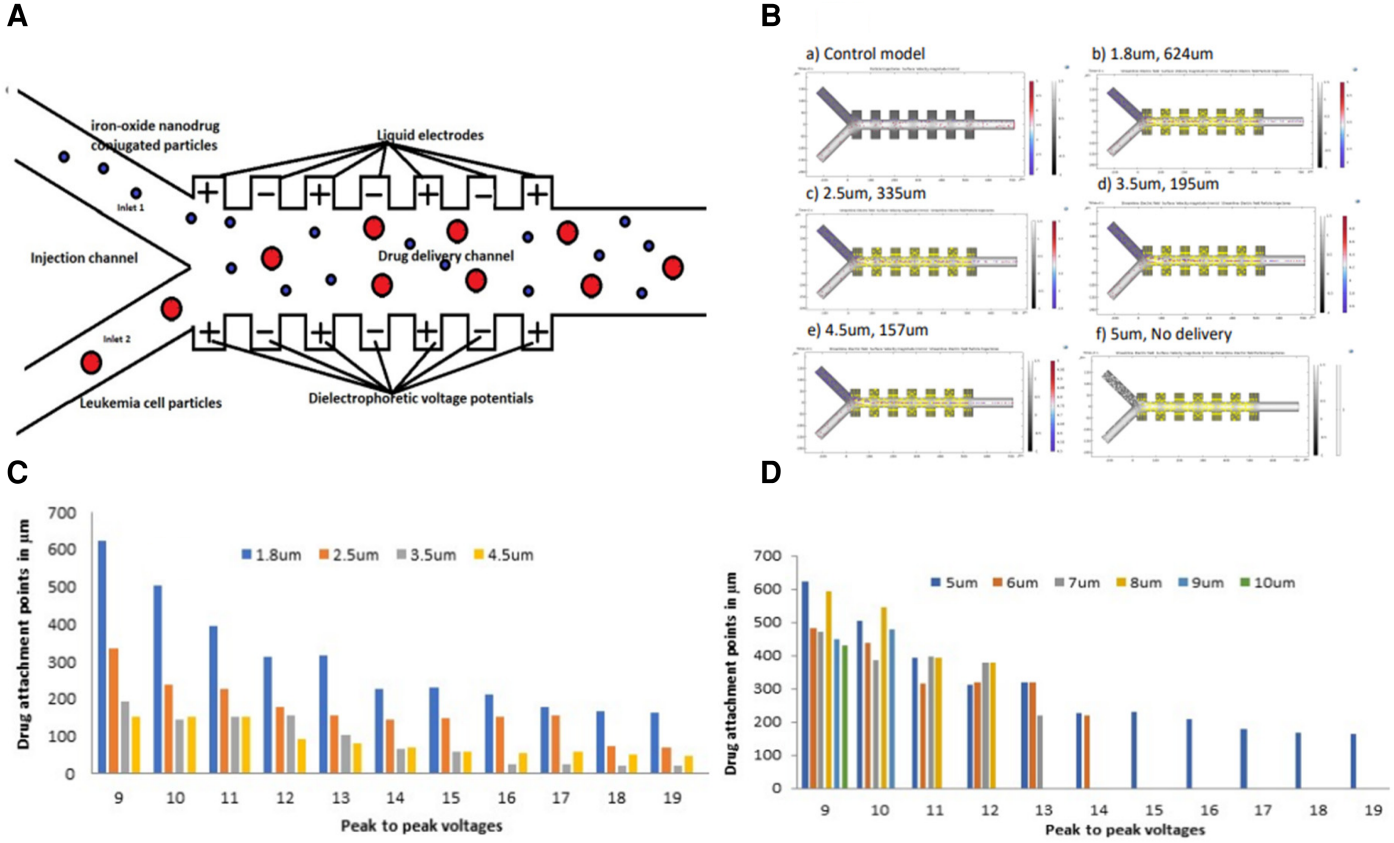
(**A**) Schematic geometry of the dielectrophoretic micro model used for drug attachment to the leukemia cells (**B**) simulations showing drug attachment points for 1.8 μm, 2.5 μm, 3.5 μm, and 4.5 μm iron-oxide particle sizes conjugated with a drug at a 9 V peak-to-peak (**C**) a histogram showing drug attachment points from the drug conjugated onto various iron-oxide particle sizes to a 5 μm leukemia cell along the micro-channel at different peak-to-peak voltages (**D**) a histogram showing drug attachment points onto various leukemia cell sizes along the micro-channel at different peak-to-peak voltages.

Figure 1A shows a proposed Y- configuration model design that can be used to deliver the anti-cancer drugs to the leukemia cells using the dielectrophoretic force. The proposed simulation model partly resembles a physical model previously fabricated for functionalizing nanoparticles (78). By convention, the left and right sides of the channel are taken in the direction the particles see while flowing. The model assumes a planar liquid electrode pattern at the bottom with dead-end chambers positioned perpendicularly to the main channel, as defined by Mernier et al. (79). Such chambers help in providing homogeneous electrical fields over the total channel height while keeping a simple process flow with a single planar metal layer. DEP signals are applied on liquid electrodes placed on both sides of the channel as partly described by Tornay et al. (80). The dielectrophoretic force attracts the leukemic cells to the micro drug particles during the movement process. The proposed model assumes a buffer flow to focus the cell towards the right side of the channel using the dielectrophoretic force. As the cells considered here have sizes larger than 500 nm, their diffusion in the attachment section can be considered to be negligible (80).

The force vectors used to calculate the trajectory of the particles are obtained by data post-processing using COMSOL software version 6.0 and calibrating the particle position with the steady-state velocity. The model uses the following physics interfaces to carry out the numerical simulations. (1) Electric Currents to model the electric field in the microchannel, (2) Creeping flow to model the fluid flow and (3) Particle tracing for fluid flow to compute the trajectories of the leukemia cells and the micro-drug under the influence of hydrodynamic focusing and dielectrophoretic forces. Our group simulated three parameters in studying the drug attachment to the leukemia cells using this model and these included; (1) Dielectrophoretic peak-to-peak voltage, (2) Leukemia cell size and (3) Iron-oxide particle size conjugated with the drug. Starting with the 5 μm leukemia cell size as our reference early-stage size of leukemia cell and 1.8 μm iron-oxide particle size conjugated with the drug, 9 Vpp was applied and the simulation ran for approximately 3 min. More voltages were applied ranging from 9 Vpp to 19 Vpp beyond which no attachment could be registered. Various leukemia cell sizes were also simulated ranging from 5 μm to 10 μm while keeping a constant 1.8 μm iron-oxide particle size and constant peak-to-peak voltage. Iron-oxide particle sizes were also simulated ranging from 1.8 μm to 5 μm while keeping a constant 5 μm leukemia cell size and constant peak-to-peak voltage. All these simulation values were performed up to a point when no drug attachment could be attained.

Attraction by positive DEP is used here in combination with hydrodynamic focusing to enable particle attachment (80)*.*

Figure 1B shows the drug attachment simulations from 1.8 μm, 2.5 μm, 3.5 μm, to 4.5 μm iron-oxide particle sizes conjugated with a drug at 9 V peak-to-peak. The simulations were also taken at a constant leukemia cell size of 5 μm. A control model clearly shows no attachment of the drug to the leukemia cells without DEP force. The simulations also indicate a general decrease in the drug attachment points from 624 μm to 157 μm with an increase in the iron-oxide particle size from 1.8 μm to 4.5 μm. Finally, the model reaches a final point with a 5 μm iron-oxide particle size from which no drug was attached to the leukemia cells.

Figure 1C shows a cross-section of drug attachment points onto a 5 μm leukemia cell using various iron-oxide particle sizes onto which a drug is conjugated. Although there was a steady decrease in the attachment points as the peak-to-peak voltages increased, all the various iron-oxide particle sizes were able to deliver the drug to the 5 μm leukemia cell.

Figure 1D shows a cross-section of drug attachment points onto various leukemia cell sizes using different peak-to-peak voltages. These results show a tremendous decrease (improved attachment efficiency) in the attachment points with an increase in the voltages. The results additionally show a drug attachment failure as the cells increased in size with an increase in the voltage potential.

## 4. Discussion

The electrical conductivity of iron-oxide particles is due to the de-localization of π-electrons along the π-conjugated backbone. Such delocalized electrons get stabilized by dopant ions to the oxidized leukemia cells and then create a continuous conduction flow. During such a flow, the drug conjugated onto the micro iron-oxide particle undergoes a redox reaction with the leukemia cells leading to a drug attachment onto this cell (81). Such drugs are mainly released by electrostatic repulsion caused by an applied potential that reduces the iron-oxide particles to attach the drug to the leukemia cells. The drug attachment rate depends on the morphology of the iron-oxide particle (including its size and density) and its electromechanical properties. The parameters used during this simulation protocol (especially the peak-to-peak voltages and the size variations) directly affect the electrical properties of the iron-oxide particles and the preceding drug attachment rate. Although other parameters like the pH and the surrounding temperatures can also affect the drug attachment patterns, the peak-to-peak voltages and the particle sizes can additionally be customized in various drug attachment studies (82). This simulation protocol similarly affects the drug attachment rate as it does the response of the particles to the electric field. The drug conjugated onto the iron-oxide particle was designed with a tiny thickness to become non-electroactive at applied voltage potentials during the drug attachment onsets (83). This tiny thickness was aimed at providing a neutrally charged drug that easily attaches to the iron-oxide particles without being limited by either the positive or the negative charges (84). Dielectrophoretic fields may have different effects on cellular structures related to pulse durations and strengths. Such fields cause the cell membrane effects to decrease while increasing the intracellular effects to enhance the attachment process (85). Furthermore, increasing the iron-oxide particle diameter with the dielectrophoretic voltage enhanced the attachment efficiency as shown in Figures 1C,D. However, in Figure 1D, due to a further increase in leukemia cell particles from 6 μm to 10 μm at a constant iron oxide micro particle size of 1.8 μm, there was no drug attachment.

The surface area to volume (SAV) ratio plays a crucial role in the attachment of iron-oxide micro particles to micro-sized leukemia cells during targeted drug simulations. In the context of drug delivery, it determines the available surface area for interactions between the particles and the cells (86, 87). A higher SAV ratio means a larger surface area relative to the volume of the particles. This increased surface area provides more sites for attachment and enhances the probability of interaction between the iron-oxide micro particles and the leukemia cells. Consequently, a higher SAV ratio can promote more effective and efficient attachment of the particles to the cells, leading to improved targeted drug delivery.

The attachment process relies on various factors, including surface chemistry, charge, and ligand functionalization of the particles, as well as the characteristics of the target cells (88, 89–91). However, assuming all other factors remain constant, a higher SAV ratio generally facilitates greater contact between the particles and the leukemia cells, increasing the likelihood of successful attachment. This phenomenon explains the reason as to why no attachment occurred between 1.8 μm iron oxide particles and leukemia cell particles greater than 6 μm. Therefore, by maximizing the SAV ratio, researchers can enhance the binding efficiency of iron-oxide micro particles to leukemia cells during targeted drug simulations, ultimately improving the efficacy of drug delivery systems designed for treating leukemia or other diseases.

Although our proposed model is computationally microfluidic, it rhymes well with physically designed models that have used DEP with whole blood samples. DEP has been studied as a method to isolate Circulating Tumor Cells (CTCs) from whole blood samples based on their different electrical properties compared to normal blood cells (92). Once isolated, these CTCs can be analyzed for genetic mutations, drug susceptibility, and other factors that can guide treatment decisions. A separation test was conducted with live K562 cells, achieving a high separation efficiency of 94.74% using the DEP force at 7 Vp-p with 10 kHz. Separation efficiencies were evaluated for treated samples at 12 and 24-h durations, achieving high efficiencies of 94.71% and 93.25%, respectively. Other researchers have employed DEP analysis to delve into the underlying mechanisms governing separation and apoptosis principles at both gene and cellular levels using different leukemia cells (93, 94). The discernible variations in cell membrane capacitance and cytoplasmic conductivity, as identified by DEP analysis, offer the potential for the early detection of apoptosis. This technology holds the promise of aiding physicians in promptly detecting apoptosis, facilitating more timely and tailored patient treatments. As the field continues to evolve, the advancement of this technology could pave the way for increasingly individualized and personalized therapeutic approaches for patients.

The cost of leukemia treatment can vary significantly depending on several factors, including the type of leukemia, the stage of the disease, the specific treatment regimen prescribed, the location of treatment, the duration of treatment, and the healthcare system of the country. Leukemia treatment costs can encompass a wide range of expenses, such as consultations, diagnostic tests, medications, hospital stays, procedures, supportive care, and follow-up appointments. It's important to note that leukemia treatment costs can be substantial, and they can create financial burdens for patients and their families. Although different attempts have been implored to reduce the leukemia treatment costs (25, 95–98), the overall prices may still be unaffordable to the LMICs, hence necessitating cheaper and affordable options. While our proposed model may not directly lower the costs of medications or medical procedures, it can help make the treatment process more efficient and targeted, potentially leading to cost savings in the long run. The setup involves acquisition of a one-time strong computer that could cost approximately $1,500, COMSOL yearly software license and modules that could cost $3,500. Such a setup could support as many patients as possible and could provide pre-treatment guidance to the Physicians.

## 5. Conclusion

We have computationally developed a simulation model able to deliver micro-drugs to the early-stage leukemia cells. The device uses a combination of flow focusing and DEP to deliver these drugs to the cells depending on their size. The electrical field and flow speed have been calculated by numerical simulations. Furthermore, the use of relatively low voltages makes it suitable for point-of-care applications. In a broader perspective, the proposed model can be used to deliver micro-drugs to other cancer cell types featuring similar differences in size. Such a model provides a favorable discussion about the efficacy, safety and the affordability plans of the therapy before implementing it to the patient.

## Data Availability

The original contributions presented in the study are included in the article/Supplementary Material, further inquiries can be directed to the corresponding author.
